# Pharmaceutical Wastewater Effluent—Source of Contaminants of Emerging Concern: Phytotoxicity of Metronidazole to Soybean (*Glycine max*)

**DOI:** 10.3390/toxics5020010

**Published:** 2017-04-02

**Authors:** Okhumode H. Yakubu

**Affiliations:** Department of Public Health Sciences, College of Health and Social Services , New Mexico State University, Las Cruces, NM 88003-8001, USA; ohyakubu@nmsu.edu; Tel.: +1-575-650-4126

**Keywords:** Active Pharmaceutical Ingredients (APIs), contaminant of emerging concern, effluent, Effluent Treatment Plant (ETP), metronidazole, Pharmaceutical Manufacturing Companies (PMCs), Relative Growth Rate (RGR), soybean (*Glycine max*), wastewater

## Abstract

Industrial discharge of active pharmaceutical ingredients (APIs) into the environment in some middle- and low-income countries is not sufficiently regulated. The phytotoxicity of metronidazole (FLAGYL)—one of the most commonly used over the counter (OTC) antibiotics, to soybean (*Glycine max*) is investigated. Relative growth rate (RGR) expressed in gram per gram per day (gg^−1^d^−1^) was applied to plants destructively harvested at maturity (42 d), to determine the toxicological impact. Differences between mean RGR of the three groups were performed at 0.05 significance level. Multiple comparisons suggest that there was a statistical significant difference among mean RGR for all treatment groups. Metronidazole is toxic to soybean plants (*Glycine max*) based on dose-response criterion. There is a need to enforce treatment of pharmaceutical wastewater effluent by Pharmaceutical Manufacturing Companies (PMCs) before discharge into the environment.

## 1. Introduction

Pharmaceutical manufacturing has until recently been generally considered to not significantly contribute to environment pollution on the one hand, and to not pose any environmental threat of consequence, on the other [1,2]. Recent findings however suggest the contrary: certain manufacturing facilities across the globe have been determined to discharge into the environment significantly high levels of APIs, beyond previously established levels, thereby causing pollution [2,3,4,5,6]. Antibiotics constitute a significant proportion of APIs found in the environment, and have become a class of contaminants of emerging concern. They have been detected at concentrations between a few ng/L to µg/ L, as well as at levels as high as mg/L range in both industrial effluents and their receiving water bodies, including surface and groundwater across various countries of the world [7,8,9,10,11,12]. High levels of the antibiotic oxytetracycline were detected at 600 mg/L in the ETP discharge of a PMC in Asia [9]. In developing nations like Nigeria, there is little or no evidence of regulatory disposal of pharmaceutical wastes, and a large number of PMCs either lack an ETP, or installed ones are non-functional [13]. Unregulated methods of waste disposal have resulted in the occurrence of APIs in high concentrations beyond tolerable levels. Undoubtedly, the implication for environmental health regarding soil and water quality is negative. Also, some authors have speculated that industrial use of pharmaceuticals is a major source of environmental pollution [14]. Moreover, studies implicating pharmaceutical ETP as a real source of pollution are well documented [7].

Food crisis in some African countries like Nigeria has resulted in elevated small scale cropping including subsistence farming. Consequently, there is increased reliance on irrigation from various non-precipitation water sources including polluted ponds, rivers, and streams [15,16]. Part of the strategy to augment rainwater is the use of industrial wastewater and their receiving water bodies. In the study area, PMCs discharge untreated effluent heavily laced with APIs including antibiotics into surrounding water bodies [16]. Small-scale soybean (*Glycine max*) production has recently experienced a significant increase, mostly to address the endemic malnutrition in affected regions. Soybean (*Glycine max*) is an economical legume, containing twice the protein content of beef and poultry, yet costing only a fifth of their price [17]. Metronidazole (2-methyl-5-nitro-1H-imidazole- 1-ethanol) belongs to the antibiotic class nitroimidazole [18]. Used for the management of both human and animal diseases of various causative organisms [19,20,21,22], it is one of the most common and widely consumed over the counter (OTC) antibiotics, thus its production in very large quantities [23,24]. Levels as high as 34 mg/L were detected in the ETP sample of a PMC in Lagos, Nigeria [1]. Metronidazole does not readily adsorb to sediments or soil, explaining its high availability and potential uptake by plants [25]. Furthermore, it has been reported to have low biodegradability in the environment [26,27,28].

To date, there is a paucity of studies addressing phytotoxicity of APIs, yet most of them focus on the subject from the stand point of soil amendments, including application of biosolids and manure to agricultural lands [29,30,31,32]. Such studies entailed use of small approximate quantities of APIs that may be obtainable in animal manure or sewage. Also, majority of these studies established the fact that legumes including soybean (*Glycine max*) were particularly sensitive to low levels of antibiotics. Among three antiprotozoal drugs tested for toxicity to soybean (*Glycine max*), metronidazole was reported to be the most toxic at low levels. Additionally, the degree of their responsiveness was determined by environmental conditions, namely soil properties. Research suggests that the environmental fate and transport of antibiotics is influenced by a number of factors: physical properties of the compound such as lipophilicity, volatility, sorption potential, and water solubility. Moreover, antibiotic soil sorption can significantly affect such vital factors as maneuverability, responsiveness to environmental elements including contaminants, as well as availability for microbial disintegration [32,33]. Soil composition including clay content, ionic strength, organic matter content, as well as pH and texture have been determined to alter the degree and mechanism of soil sorption [34].

The objective of the present study was to evaluate the phytotoxicity of approximate concentrations of metronidazole found in wastewater effluents of PMCs to soybean plants (*Glycine max*), sown in humus-rich loam soil, in order to estimate the risk involved in the use of industrial effluents as a source of irrigation water for the cultivation of the crop. It is also important to demonstrate the environmental harm pharmaceutical manufacturing poses when wastewater effluent is discharged into the environment untreated.

## 2. Materials and Methods 

### 2.1. Experimental Design

The experiment was conducted in a greenhouse (GH) at the Faculty of Science, University of Lagos (UNILAG) Lagos Nigeria. Environmental conditions of the GH were monitored at a temperature of 30 °C and relative humidity of 50%. Loamy soil rich in humus was collected from top 30 cm portion of field at a site near UNILAG. It was bulked, air dried, and sieved through a 2-mm mesh to remove pebbles and debris prior use. One kilogram of composite soil obtained from uncultivated plot was weighed into polythene bags measuring 19 × 20 cm in size. The experiment was laid out using 3 treatment groups with 5 replicate pairs per group, at 2 different times making a total of 60 bags. The seeds were sown at a seed rate of 1 seed per bag, and at a seed depth of 4 cm. A single dose (one time) application of metronidazole solution was carried out at 0 mg/L (control), 50 mg/L and 100 mg/L concentration 42 d after sowing. The spiked water was applied at the base of the plants on the soil surface. Growth parameters measured were carried out after destructive harvest at 5 d and 10 d post treatment. 

Following antibiotic exposure, subsequent irrigation was carried out with deionized water until destructive harvest was performed. The pairing method [35] was adopted in estimating the RGR. This involved grouping into pairs equally sized individuals prior to antibiotic exposure. Single plants from each pair were harvested on the first harvest date, and the other, on the second date. RGR was then calculated for each pair following destructive harvest.

For the purpose of this study, individual plants from each pair of each treatment group were harvested 5 d post treatment, and the other plant of the same pair, 5 d after the first destructive harvest that is, 10 d after treatment. All other experiments including weighing and analytical tests were carried out in the chemical lab of Vitabiotics Lagos, Nigeria.

### 2.2. Chemicals and Materials

Metronidazole (CAS Number: 443-48-1, ≥99% pure) was obtained from Meyer Organics, Mumbai, India. The distilled water (prepared in-house) was obtained from Vitabiotics Oregun, Lagos, Nigeria.

Soybean TGXI485-ID variety was obtained from the International Institute of Tropical Agriculture (IITA), Ibadan, Oyo State, Nigeria.

### 2.3. Soil Analysis

The sieved humus-rich loamy soil collected from the top 30 cm portion of field at a site near UNILAG was used for analysis. This soil type is recommended for optimal growth and development of soybean (*Glycine max*) [36].

#### 2.3.1. Soil pH

The soil pH was determined potentiometrically in a 1:2 soil to water ratio slurry using an electronic pH meter [37]. An Elico LI 120 pH meter was used. The pH was determined before sowing and at the end of the second destructive harvest. A standard pH buffer 7.0 tablet was dissolved in 50 mL of distilled water in a 100-mL beaker, using a glass rod and then stirring on a Gallenkamp magnetic stirrer with a magnetic bar in the beaker. The solution was then transferred into a 100-mL volumetric flask and made up to mark. The Elico LI 120 pH meter was calibrated using the standard pH buffer 7.0 solution. Five grams of the sieved soil was scooped into a 50-mL beaker to which 10 mL of distilled water was added. The solution was stirred vigorously for 15 s and allowed to stand for 30 min. The electrode was placed in the slurry and swirled carefully, and the pH was immediately read. 

#### 2.3.2. Soil Organic Matter Content

The loss-on-ignition (LOI) method was employed. It is a semi-quantitative method based on the indiscriminant removal of all organic matter. 

A ceramic crucible was heated at 390 °C and cooled in a desiccator. The process was repeated until a constant weight was obtained. About 2 g of the soil sample was placed in the weighed crucible and heated at 390 °C for 24 h [38,39]. The sample was cooled and weighed. Organic matter content was calculated as the difference between the initial and final sample weights divided by the initial sample weight times 100%. The process was repeated and the results obtained were averaged. Weighing was done using a citizen model CY204 chemical balance whose capacity was 210 g maximum. A Gallenkamp muffle furnace was used for igniting the soil sample. The organic matter content of soil was calculated as weight of residue/weight of sample before ignition × 100%.

### 2.4. Seed Planting, Labelling, and Treatment

The seeds were sown in labelled black polythene bags containing 1 kg of soil. The bags were arranged in rows 30 cm apart and about 5 cm between each row in the GH. The seeds were sown at a depth of 4 cm and at a seed rate of 1 seed per bag. After germination, which was between 4–7 d, wetting was carried out at 2-d intervals for the first 2 weeks, and there after increased to every 24 h. It was done either in the morning or at sunset. However, caution was taken not to cause water logging by applying only a 100 mL of water per bag.

Plants were assigned into 3 treatment groups, namely 0 mg/L, 50 mg/L, and 100 mg/L groups. Each replicate pair for all 3 treatment groups was labeled in alphabetical order, with each pair bearing similar alphabets but distinguished by subscript numbers. Plants assigned for harvesting 5 d (*t*_1_), post-treatment, were labeled with an alphabet associated with subscript 1, while those assigned for harvesting 10 d (*t*_2_) post-treatment were labeled with corresponding alphabet but associated with subscript 2. Based on the Evans method [35], this design implies that for each treatment group i.e., 0 mg/L, 50 mg/L, and 100 mg/L, there were A_1_, A_2_ pair; B_1_, B_2_ pair; C_1_, C_2_ pair; D_1_, D_2_ pair; E_1,_ E_2_ pair; F_1_, F_2_ pair; G_1_, G_2_ pair; H_1_, H_2_ pair; I_1_, I_2_ pair; and J_1_, J_2_ pair; with each pair representing similarly sized plants (10 individuals for time *t*_1_ and time *t*_2_ each) across all 3 treatment groups. The result is a total of 5 pairs at *t*_1_, and 5 pairs at *t*_2_ for each treatment group, totaling 30 pairs for all treatment groups at both times. So, for each treatment group, plants to be harvested at *t*_1_ post-treatment were labeled A_1_, B_1_, C_1_, D_1_, E_1,_ F_1_, G_1_, H_1_, I_1_, and J_1_. The corresponding duplicate pairs to be harvested at time *t*_2_, post- treatment were similarly labeled i.e. A_2_, B_2_, C_2_, D_2_, E_2_, F_2,_ G_2,_ H_2,_ I_2,_ and J_2_ (Table 1). In all, there was a total of 20 bags per treatment group, 20 bags for *t*_1_ and *t*_2_ each for control, 50 mg/L and 100 mg/L treatment group each.

Pairing similarly sized plants [35] is important in preventing bias in the experiment. Individual plants harvested at *t*_2_ provide information on change that may likely have occurred to the plant since *t*_1_.

The labeled plants were treated with 100 mL of the corresponding concentration of metronidazole and left for 24 h for complete absorption. Treatment was carried out at the flowering stage which was about 42 d from sowing. The solution was carefully applied around the root area on the soil surface.

### 2.5. Harvesting, Drying, and Weighing

Destructive harvesting was carried out 5 d (*t*_1_) and 10 d (*t*_2_) after treatment. At *t*_1_ and *t*_2_ each pair of the labeled plants from each treatment was gently pulled out whole from each bag. The soil around the roots was carefully shaken off, and the roots dabbed in distilled water before drying. 

The harvested plants were dried in a LEEC oven at 72 °C for 24 h [35]. The dry biomass of each plant was determined by weighing its constituent parts including the leaves, stem, buds, flowers and roots. These were then pooled together to get the total weight of the plant’s biomass.

### 2.6. Relative Growth Rate (RGR) and Statistical Analysis

#### 2.6.1. RGR

RGR is the rate of growth from the initial weight at 5 d to the final weight at 10 d post treatment, expressed in grams per gram per day (gg^−1^d^−1^). The total weight (*W*_1_) obtained at *t*_1_, as well as total weight (*W*_2_) obtained at *t*_2_, for the dry biomass of all replicates in each treatment category, namely, control, 50 mg/L, solution and 100 mg/L solution, were recorded.

RGR for replicates at times *t*_1_ and *t*_2_ was determined by the formula:

(1) $$\mathrm{RGR}=\frac{\ln\;W_{2}\;-\;\ln\;W_{1}}{t_{2}\;-\;t_{1}}$$

where *W*_2_ = weight of dry biomass at final harvest (10 d post treatment); *W*_1_ = weight of dry biomass at initial harvest (5 d post treatment); *t*_2_ = final harvest date (10 d); *t*_1_ = Initial harvest date (5 d).

#### 2.6.2. Statistical Analysis

**Figure 1:**
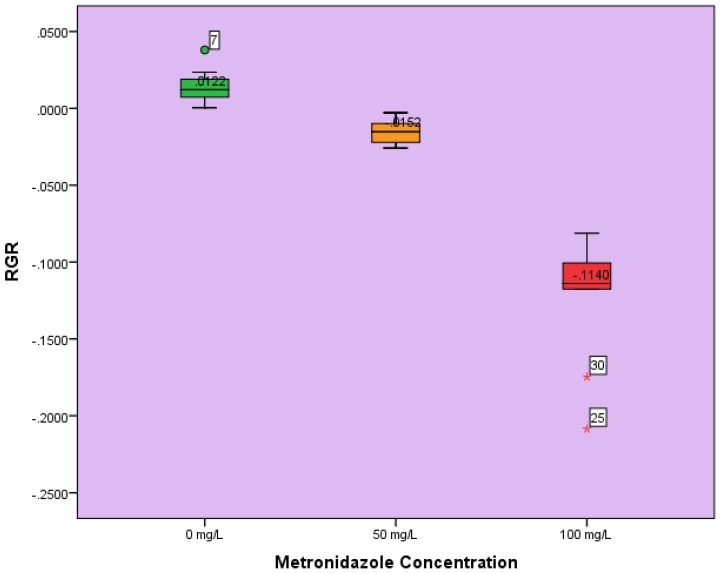
Box plots obtained for all treatments. It provides a visual representation of the impact of metronidazole on the relative growth rate (RGR) of the plants.

The results obtained from weighing plant dry biomass were subjected to statistical evaluation. IBM SPSS 24 was used to perform all the statistical analyses presented in the results section. The descriptive statistics including mean and standard deviation (SD) were calculated for each treatment group. The relationship between spiked concentration of metronidazole applied to the plants, and the consequent toxicity as expressed by RGR, was quantified. A box plot (Figure 1) was generated from the individual RGR for each plant vs. spiked concentration of metronidazole, to illustrate the differences in the distribution of RGR within individual group, and among the 3 different treatment groups. One-way ANOVA was used to test the differences in the mean RGR obtained for the different treatments. Additionally, with a Tukey’s Honestly Significant Differences (HSD), multiple comparisons were conducted to determine if the pairwise differences between the mean RGR for the 3 treatments groups were statistically significant. All the tests were performed at a significance level 0.05. Mean RGR for groups in homogeneous subsets were also determined using HSD.

## 3. Results

### 3.1. Soil Analysis

Table 2 presents results obtained for pH test conducted on soil samples from all the bags after the final harvest. The pH obtained for the bulk soil before seeds were sown was 6.49.

The mean organic content obtained for the bulk soil sample was 19%. Soil organic matter greater than 3.5% is adequate for plant growth [40,41].

### 3.2. RGR

For ease of computing data, the alphabets were replaced with Arabic numerals so that A_1_ B_1_ C_1_ D_1_ E_1 …_ I_1_ and A_2_ B_2_ C_2_ D_2_ E_3 …_ I_1_ were represented in the tables by 1, 2, 3, 4, 5, 6, 7, 8, 9, 10, respectively, for all treatment groups (Table 3, Table 4 and Table 5). 

Within 24 h of treatment, the impact of the application was observed in the treated plants by rapid physical changes: Plants in the 100 mg/L treatment group showed drastic change evidenced by droopy leaves. Droopy leaves were not observed among the 50 mg/L treatment plants, however, at 5 d, they had become slightly yellow, while plants in the 100 mg/L treatment group were rapidly wilting at the time. At 10 d, practically all the plants in the 100 mg/L treatment group had wilted, and the yellowing of leaves deepened for the 50 mg/L treatment group.

All plants in the control group flourished with lush leaves. Flowering and seeding were also observed among plants in this group. 

The total weight (*W_1_*) obtained at 5 d (*t*_1_) after treatment, as well as total weight (*W*_2_) obtained at 10 d (*t*_2_), for the dry biomass of all replicates in each treatment group, namely control, 50 mg/L, and 100 mg/L, are shown in Table 3, Table 4 and Table 5 respectively. 

For the control, between 5 d and 10 d, all replicates showed an increase in weight. Replicates treated with 50 mg/L and 100 mg/L of the antibiotic all showed a decrease in weight between 5 d and 10 d, weight loss being more severe in replicates treated with 100 mg/L of the spiked solution (Table 3, Table 4 and Table 5).

### 3.3. Statistical Analysis

Figure 1 shows the box plots obtained for all treatments. It provides a visual representation of the impact of metronidazole on the RGR of the plants.

The performance of one-way ANOVA applied to all the data categorized by the spiked concentrations revealed significant differences in RGR (*p* < 0.0001) between control and treated plants. The results from multiple comparisons suggest that the difference between control and 50 mg/L treatment was statistically significant (*p* < 0.025); between control and 100 mg/L treatment was also statistically different (*p* < 0.0001); as well as the difference between 50 mg/L and 100 mg/L (*p* < 0.0001). Table 3, Table 4 and Table 5 also present the phytotoxicity of metronidazole to soybean (*Glycine max*) when carefully selected plants of the legume were exposed to approximate concentrations of the antibiotic found in industrial effluents. Replicates in the 100 mg/L treatment group were more severely impacted than the 50 mg/L group. 

Table 5 shows a negative growth (*W*_2_ < *W_1_*) for all replicates in the group, indicating antibiotic poisoning. The mean RGR obtained was −0.122 ± 0.039. For the 50 mg/L treatment group, all the plants were also negatively affected following exposure. Table 4 illustrates the negative growth observed (*W*_2_ < *W_1_*). RGR obtained was −0.016 ± 0.007. Table 3 shows that all unexposed replicates were unaffected (*W*_2_ > *W*_1_). RGR obtained was 0.014 ± 0.011. These results indicate that metronidazole is toxic to replicates in both exposed groups (50 mg/L and 100 mg/L) by retarding plant growth. However, the toxicity observed in the 100 mg/L treatment group was more severe than the 50 mg/L treatment group (higher negative value for RGR: −0.122 ± 0.039 > −0.016 ± 0.007). The mean RGR obtained for both exposed groups suggests approximately, an eight-fold difference in wilting or growth retardation between the 50 mg/L and 100 mg/L treatment groups.

The box plots (Figure 1) further support results shown in Table 3, Table 4 and Table 5, and those obtained from multiple comparisons. The distribution of RGR among replicates in the 100 mg/L group was more skewed towards the upper percentile, with a median of −0.0114. This indicates that although the antibiotic poisoned all the plants, a few were more severely impacted than others were, within the treatment group. The RGR distribution of replicates in the 50 mg/L treatment group with a median of −0.0152 was fairly even, i.e., the phytotoxicological impact on most of the plants was the same as indicated in the box plot. The box plot for the control group with a median of 0.012 also shows a fairly even RGR distribution, although a few more of the plants increased in size than others within the group. The mean RGR for groups in homogeneous subsets indicates that none of the treatment groups were in the same category in terms of significant difference. In other words, mean RGRs for all groups were statistically significantly different.

## 4. Discussion

### 4.1. Soil Analysis

The optimum soil pH for soybean cultivation is between 6.0 and 7.5 [40,41].

Table 1 shows on average a decline in soil pH in the order: control > 50 mg/L > 100 mg/L, the mean values of the pH being 7.04 > 6.82 > 6.49 respectively. This may have different implications. First, for control and 50 mg/L treatment group, the rise from the initial pH of 6.49 obtained at pretreatment may be due to the fact that watering of the soil brought about the dissolution of several substances and altered the soil chemistry causing the pH to rise, tending more towards the neutral side. However, the impact of metronidazole on the soil pH is still observable in the mean values obtained for the 50 mg/L and 100 mg/L treatments. A saturated solution of metronidazole has a pH of 5.8 [42]. This is slightly acidic and may account for the higher acid value obtained for the higher concentration (100 mg/L) and the lower acid value for the lower concentration (50 mg/L). Second, wilting of the antibiotic-treated plants may not have anything to do with soil pH as the values obtained for all treatments were within the optimum value that has the potential of sustaining the plants’ growth [40,41]. 

The soil organic matter did not seem to interfere with the growth of the plants. A 19% organic matter was far above the requirement of 3.5% for plant growth and development.

### 4.2. RGR, Statistical Analysis and Dose Response

The results clearly show that for the control, growth was not impaired at all, as *W*_2_ > *W_1_*. However, the reverse is the case for plants treated with metronidazole solution. For these plants, growth was hampered, as *W*_2_ < *W*_1_, the extent of retardation being proportional to the antibiotic concentration applied [43].

The values obtained further buttress the impact of metronidazole on the growth of soybean plant (*Glycine max*). The treated plants withered drastically. 

Additionally, the mean RGR values for the control plants indicated an increase in weight in the dry biomass of the replicates from *t*_1_ to *t*_2_; however, mean RGR values for the antibiotic-treated plants were all negative-the higher concentration, i.e., 100 mg/L giving higher negative values than the 50 mg/L treatment. This shows that plants that took in most, showed the greatest wilting [43]. Also, the growth of soybean plants (*Glycine max*) was affected by increasing concentrations of metronidazole [29].

A few previous studies investigated poisoning of leguminous plants, including soybean (*Glycine max*) by different antibiotics. In some cases, the antibiotic killed the plants, while in others they only retarded plant growth [29,44,45]. In the present study, the phytotoxicity of high concentrations of metronidazole to exposed soybean (*Glycine max*) was explored. Toxicological effects of metronidazole increased with increasing concentration—relative growth rate decreased with increasing metronidazole concentration, in the order: RGR control > RGR 50 mg/L > RGR 100 mg/L (Figure 1).

Batchelder [44,45] applied high concentration (160 mg/L) of animal growth-promoting antibiotics, chlortetracycline and oxytetracycline to corn (*Zea mays* L. var. Grand Valley hybrid SX 121), pinto bean (*Phaseolus vulgaris* L. var. University of Idaho 114), radishes (*Raphanus sativus* L. var. White Icicle), and wheat (*Triticum aestivum* L. var. Fielder). While the legume, pinto bean (*Phaseolus vulgaris*) was affected by the exposure, the three other crops were unaffected. Additionally, whereas pinto bean (*Phaseolus vulgaris*) planted in sandy loam soil polluted with either antibiotic in separate replicates were poisoned, those planted in clay soil polluted with the same antibiotics showed no ill effects. In contrast to the unexposed group, pinto bean plants (*Phaseolus vulgaris*) grown in sandy loam absorbed smaller quantities of plant nutrients from the soil and sustained less nitrogen fixation; they were smaller, weighed less, and fructified smaller bean yields. A plausible explanation of this observation could be that the drug molecules adsorbed to the clay soil, and so pinto bean plants (*Phaseolus vulgaris*) were inhibited from absorbing the antibiotics from the soil [44,45]. He concluded that the extent of antibiotic toxicity to plant growth is strongly influenced by plant sensitivity to the contaminant drug, and the soil typical features [45]. Research suggests that environmental fate and transport of antibiotics is influenced by a number of factors: physical properties of the compound, such as lipophilicity, volatility, sorption potential, and water solubility. Moreover, antibiotic soil sorption can significantly affect such vital factors as maneuverability, responsiveness to environmental elements including contaminants, as well as availability for microbial disintegration [33,46,47]. Soil composition including clay content, ionic strength, organic matter content, as well as pH and texture, have been determined to alter the degree and mechanism of soil sorption [48]. The research methodology employed in Batcheldar’s study in terms of the concentration of antibiotics applied to the treatment groups, compares to that used in the present study. However, in contrast to the latter, which involved polluting the soil with metronidazole at the onset of plant maturity (42 d), application of the spiked antibiotics solution was performed while the soil was being air dried that is, prior to seed planting. This implies that antibiotic exposure occurred right from planting to seedling emergence through harvesting. Similar to other previous studies, the focus was on soil enrichment by manure or sewage application. Nonetheless, the findings from this study in terms of phytotoxicity also to some extent compare with observations made in the present study. Although Batchelder reported that pinto bean plants (*Phaseolus vulgaris* L.) were stunted by the antibiotics, they were not killed by the drugs as they all grew to maturity and blossomed [45]. In the present study, friable humus-rich loam soil was used as the growth medium, possibly accounting for the phytotoxicity observed, though in very significant disparity: phytotoxic effects were observed within 24 h after treatment especially for the 100 mg/ L treatment group. Here, a plausible explanation could be the difference in soil composition. Although soils used in both studies were loamy in nature, there was a significant difference in organic matter content: 0.80% and 19% for the previous and present study respectively. Given the high organic matter content of the soil used in the present study, the texture, which is also an influencing factor in the mobility, availability, and reactivity of antibiotics, may have been altered in such a way that the clay content was significantly reduced below that in the previous study (12%), thus affecting other properties and functions of the soil. The pH of both soils were close. Although prior treatment, soil pH in the present study was 6.49, mean pH slightly increased (Control: 7.04; 50 mg/L: 6.82) or remained the same (100 mg/L: 6.49) following introduction of spiked antibiotic solution. These values are close to that (7:00) obtained for soil in the previous study. However, it is vitally important to note at this point that following application of spiked solutions of either chlortetracycline or oxytetracycline, soil pH dropped to 5.1, resulting in a significant difference in pH for each of the treatment group in the present study. The required pH for optimum growth of soybean (*Glycine max*) is 6.0 to 7.5 [41]. The soil pH may have interfered with the overall result obtained in the previous study. A major difference in the methodology is the fact that while in Batchelder’s GH experiments, the spiked solution was applied while soil was being stirred and air dried, in the present study however, application was carried out by pouring the antibiotic solution on the soil surface at 42 d. It is uncertain if this has an impact on the phytotoxicity difference observed in both studies. It may be possible that for the previous study, stirring sufficiently homogenized the soil with the spiked solution of the antibiotics, consequently diluting their concentration throughout the medium, and reducing the quantity available to the roots of the plants at any given time throughout the entire growth period. The phytotoxic effects obtained for both chlortetracycline and oxytetracycline on the legume were similar. Plants that were not sufficiently poisoned or killed by the uptake of antibiotics have the potential of transferring the absorbed pollutant from the soil to the food chain [44]. It is noteworthy that in the present study, apart from the fact that the experiments were carried out in a GH, a typical farming situation was simulated. Farmers either make channels on their farmlands and convey water for irrigation from polluted sources, or fetch water in containers from the sources and sprinkle on their crops [49]. 

Another study compared the toxic effects of three pharmaceuticals, namely chloroquine, quinacrine, and metronidazole on soybean (*Glycine max*) [29]. Observations from this study suggest that the GH seedlings showed ill effects at 13 d after sowing. Moreover, emerged seedlings were particularly sensitive to as low as 0.5 mg of metronidazole per gram soil. On the other hand, concentrations of as high as 8 mg of chloroquine per gram soil, and 10.6 mg of quinacrine dihydrochloride per gram soil were required to exert any toxicological impact on the seedlings [29]. Although this study like the present one addresses phytotoxicity of metronidazole to soybean (*Glycine max*), there are major differences between both studies, in terms of objectives, experimental design, and, to a certain extent, the materials used. While the present study like Batchelder’s GH experiments focused on approximate concentrations of metronidazole found in the effluents of PMCs [44,45,46], Jjemba’s experiments investigated concentrations that may be introduced to farmlands through soil amendments [29]. In both Jjemba’s and the present studies, phytotoxicity was observed for metronidazole treated soybean (*Glycine max*). However, it is not clear how the reported concentrations were obtained = Jjemba alleged that “aliquots of the test substances were applied to the soil to obtain the required concentration”. Concentrations were consequently reported in milligram of antibiotic per gram of soil [29]. The method described may not justify the antibiotic claim, unlike Batchelder’s and the present study where application method was elaborately described. In Jjemba’s study it is uncertain if the test substances were homogenized with the soil samples as in Batchelder’s experiments [45], or if the aliquots were applied on the soil surface close to the base of the plants, as in the present study. In terms of materials, while Jjemba [29] employed Palmyra gravelly silt loam with an organic matter content of 4.9%, the current study involved a humus-rich loam with an organic matter content of 19%. There is certainly a huge difference between both soils. It may be assumed that Palmyra gravelly silt differs from the soil used in the present study in terms of properties such as texture, as the name suggests. Also, pH was reported as 6.6%. It is unclear if the ‘%’ was a mere typo, or it was meant to represent some other number, or the information was entirely wrong. pH has no units and cannot be expressed in percent. Jjemba’s study accessed toxicity by mere visual examination. In the present study however, toxicity was further examined by evaluating RGR of destructively harvested plants at 42 d [35]. The harvested plants were dried and weighed, and data obtained were subjected to statistical analysis to determine the extent of dose-response, as well as the extent of poisoning of exposed groups compared to the unexposed group. Additionally, while the previous study used a small sample size of three replicates per group, the present study explored 10 replicates per treatment, so as to increase the chances of observing any significant difference in phytotoxicity. All things being equal, in Jjemba’s study, a larger sample size may have produced a different result for the 0.5 mg g^−1^, which claimed 66.7% of the exposed plants exhibited stunted growth, and was reported as the least concentration for phytotoxicity of metronidazole to the seedlings [29]. Moreover, like Batchelder’s experiments which differ from the present study in terms of method of antibiotic application, Jjemba’s GH experiment also differed in this regard [29]. 

In order to base the observation made by Jjemba [29] on direct, rather than indirect (complete eradication of soil microbes), phytotoxicity, Migliore et al. [43] performed similar studies in an aseptic artificial growth medium. Their findings also suggest that antibiotics including sulphadimethoxine were toxic to the plants. Based on these findings, the researchers concluded that the growth and development of plants were dependent on both the properties of the growth medium and the concentration of the pollutants they are exposed to [43,44]. The disparities in the observation made between both previous studies and the present study may be influenced by various factors already highlighted. For instance, a question might be raised as to why seedlings in Jjemba’s studies were killed, unlike seedlings in Batchelder’s studies that sprouted and blossomed, even though some phytotoxicity was observed. Plant’s toxicological response depends on the sensitivity of the plant as well as the soil’s characteristics. Additionally, others report that in addition to soil properties, plant growth and development depend on the nature of contaminant they are exposed to [43].

In comparison with the present study, none of the previous studies took into consideration the pH of the soil after harvesting, to ascertain possible interference by change in soil pH due to antibiotic application. The current study however, considered this aspect important and investigated pH following the final destructive harvest. Soil pH for individual bags was still within recommended range for proper growth and development of soybean (*Glycine max*). On the whole, the present study is the first in recent times to assess the phytotoxicity of metronidazole to soyabean (*Glycine max*) by measuring RGR, and focusing on exposure of plants from the perspective of PMCs effluent discharge. Furthermore, a typical farming operation in terms of introduction of contaminant APIs is simulated. Considering the fact that PMCs wastewater may be applied at any point in the developmental stage of the plant, it was important to investigate what happens when the plants were exposed at their prime, hence accessing phytotoxicity at 42 d. In spite of the fact that researchers in the previous studies presented noble experiments, it is undetermined if farmers cultivate their lands by mixing up the manure in a way that the concentrations reported are attained in real-life farming operations. These studies require further studies, emphasizing simulation of real farming operations. High concentrations obtainable in the wastewater discharges of pharmaceutical manufacturing operations and processes were represented in the present study.

### 4.3. Uptake and Phytotoxicity

Based on the findings from several studies, Carvalho et al. [50], in their review of plant-pharmaceutical interactions, suggest that both pharmaceutical-induced toxicity in plants, and the nature and extent of observed toxicological effects, are dependent on the plant species, as well as the type and concentration of the API they are exposed to. In Batchelder’s studies where plant mortality of pinto beans (*Phaseolus vulgaris*) increased with increasing concentration of contaminant antibiotics, he hypothesized that the antibiotics were directly responsible for the observed phytotoxicological effects. His research also speculates the possibility of direct or indirect phytotoxicity by degradation products, or calcium deficiency arising from complexation with calcium ions [45]. Farska et al. [51] suggest that calcium chelation in the leaves of pinto bean (*Phaseolus vulgaris*) was a plausible phytotoxic mechanism, when in their experiments 3 H-labelled tetracycline was found within the leaves of the exposed plants. Kong et al. [52] in their own study, hypothesized that ill effects by oxytetracycline to exposed alfalfa (*Medicago sativa*) was due to translational activity impediment of chloroplast and chloroplast (p)ppGpp synthase activity. In other words, phytotoxic effects may be triggered by disruption or modification of enzyme activity. Furthermore, decrease in photosynthetic pigments including chlorophyll and carotenoids, and the consequent reduction in plant content of fatty acids, flavonoids, phenols, protein, and sugars, have been associated with phytotoxicity [53,54,55]. Phytotoxicity may also be as a result of bioaccumulation of the pollutant antibiotics in the plants leading to a decrease in the supply of folic acid. Folic acid plays a very significant role in the production of purines, which are essential precursors in the biochemical routes of cytokinins and abscisic acid [43].

Although in the present study, the mechanism of observed phytotoxicity was not investigated, I hypothesize that any of the forgoing observations made by authors in previous studies is a likely phytotoxic mechanism.

## 5. Conclusions

This research investigated the phytotoxicity of metronidazole to soybean (*Glycine max*), a highly economical crop, through exposure by the use of untreated process water from PMCs effluent as irrigation water. Pharmaceutical waste constitutes an environmental nuisance. They also have detrimental effects on diverse ecosystem fauna and flora. It is possible that higher than reported concentrations of APIs are sometimes present in the immediate environment, particularly where such practices as flushing untreated solid wastes directly into the drains, indiscriminate disposal in surrounding areas, burying in the ground, and discharging untreated wastewaters into drains and farmlands, are perpetrated.

The findings from this study have vital implications to a diverse set of stakeholders including environmentalists, regulating agencies, PMCs and more importantly, farmers. APIs including antibiotics can be introduced into the environment in various ways. In recent times when industrial wastewater or receiving water usually containing very high quantities of APIs, are being used as alternative sources of irrigation water for crops, there is need for responsible regulating agencies to enforce installation of functional ETPs to treat wastewater before discharge into the environment. It is also important for environmental agencies to set limits on the quantity of APIs that may be contained in the treated water. This requires adequate monitoring of PMCs by the responsible regulators. Farmers must be informed of the potential risk involved in the use of water as irrigation water from such sources.

Although this study entailed detailed evaluation, there is still need to conduct follow up studies entailing the investigation of a wider range of API concentrations, not only in the GH, but also on the field as well.

## Figures and Tables

**Table 1:** Similarly sized plants selected and paired prior treatment assigned to each column.

Harvesting	Replicates									
**5 d**	A_1_	B_1_	C_1_	D_1_	E_1_	F_1_	G_1_	H_1_	I_1_	J_1_
**10 d**	A_2_	B_2_	C_2_	D_2_	E_2_	F_2_	G_2_	H_2_	I_2_	J_2_

**Table 2:** Soil pH post-harvest.

S. No.	1	2	3	4	5	6	7	8	9	10	Mean
**Control**	7.00	7.10	7.06	7.12	7.00	6.95	7.24	6.89	6.94	7.00	7.04
**50 mg/L**	6.58	6.96	6.82	6.96	6.80	6.86	6.81	6.98	6.75	6.58	6.82
**100 mg/L**	6.54	6.58	6.74	6.75	6.72	6.56	6.14	6.16	6.23	6.57	6.49

**Table 3:** RGR for control plants.

Replicates	*W*_1_	*W*_2_	ln *W*_1_	ln *W*_2_	ln *W*_2_ − ln *W*_1_	RGR
1	1.2113	1.3624	0.1917	0.3092	0.1175	0.0235
2	1.2393	1.3621	0.2145	0.3090	0.0945	0.0189
3	1.4584	1.5577	0.3773	0.4432	0.0659	0.0132
4	1.4432	1.5548	0.3669	0.4413	0.0744	0.0149
5	1.2567	1.3143	0.2285	0.2733	0.0448	0.0089
6	1.2746	1.3058	0.2426	0.2668	0.0242	0.0048
7	0.8185	0.9900	−0.2003	−0.0101	0.1902	0.0380
8	0.9560	1.1059	−0.0450	0.1007	0.0557	0.0111
9	1.1130	1.1547	0.1071	0.1438	0.0367	0.0073
10	1.1643	1.1665	0.1521	0.1540	0.0019	0.0004

**Table 4:** RGR for 50 mg/L treatment plants.

Replicates	*W*_1_	*W*_2_	ln *W*_1_	ln *W*_2_	ln *W*_2_ − ln *W*_1_	RGR
1	0.8760	0.8639	−0.1324	−0.1463	−0.0139	−0.0028
2	0.8758	0.8248	−0.1326	−0.1926	−0.0599	−0.0119
3	0.8429	0.7410	−0.1709	−0.2998	−0.1288	−0.0258
4	0.8429	0.7683	−0.1709	−0.2636	−0.0927	−0.0185
5	0.7830	0.7248	−0.2446	−0.3219	−0.0772	−0.0154
6	0.7509	0.7162	−0.2865	−0.3338	−0.0473	−0.0095
7	0.6708	0.6006	−0.3993	−0.5098	−0.1105	−0.0221
8	0.6903	0.6404	−0.3706	−0.4457	−0.0750	−0.0150
9	0.8004	0.7619	−0.2226	−0.2719	−0.0493	−0.0099
10	0.8488	0.7491	−0.1639	−0.2889	−0.01250	−0.0250

**Table 5:** RGR for 100 mg/L treatment plants.

Replicates	*W*_1_	*W*_2_	ln *W*_1_	ln *W*_2_	ln *W*_2_ − ln *W*_1_	RGR
1	0.6726	0.4482	−0.3966	−0.8025	−0.4059	−0.0812
2	0.6684	0.4292	−0.4029	−0.8458	−0.4430	−0.0886
3	0.6613	0.3784	−0.4135	−0.9718	−0.5583	−0.1117
4	0.6317	0.3509	−0.4593	−1.0473	−0.5880	−0.1176
5	0.6233	0.3541	−0.4727	−1.0382	−0.5655	−0.1131
6	0.6176	0.3441	−0.4819	−1.0668	−0.5849	−0.1170
7	0.5415	0.3315	−0.6134	−1.1041	−1.0428	−0.2086
8	0.5765	0.3245	−0.5508	−1.1255	−0.5747	−0.1149
9	0.5127	0.3100	−0.6681	−1.1712	−0.5031	−0.1006
10	0.5040	0.2103	−0.6852	−1.5592	−0.8740	−0.1748
